# Predictive Value of the Naples Prognostic Score for One-Year Mortality in NSTEMI Patients Undergoing Selective PCI

**DOI:** 10.3390/diagnostics15050640

**Published:** 2025-03-06

**Authors:** Mesut Gitmez, Evren Ekingen, Sueda Zaman

**Affiliations:** 1 Department of Cardiology, Batman Training and Research Hospital, 72500 Batman, Turkey; 2 Department of Emergency, Mamak State Hospital, 06100 Ankara, Turkey; evren23@gmail.com (E.E.); drsuedazaman@gmail.com (S.Z.)

**Keywords:** acute myocardial infarction, the Naples prognostic score, percutaneous coronary intervention, mortality

## Abstract

**Objectives:** Non-ST-elevation myocardial infarction (NSTEMI) is a common and severe condition that requires rapid and accurate risk assessment and treatment. The Naples prognostic score (NPS) is a novel risk score that integrates nutritional and inflammatory parameters. The aim of this study was to investigate the NPS as a predictor of one-year mortality in NSTEMI patients undergoing percutaneous coronary intervention (PCI). **Methods:** This retrospective study included 197 NSTEMI patients who underwent selective PCI from January 2020 to December 2020. The NPS was calculated based on the total cholesterol, serum albumin, neutrophil/lymphocyte ratio, and lymphocyte/monocyte ratio. Patients were categorized into two groups based on their NPS values: Group 1 (NPS 0–2) and Group 2 (NPS 3 or 4). The one-year mortality status of the patients was determined through phone calls or by querying the national death registry system. **Results:** During the follow-up period, the overall mortality rate was 19.3% (*n* = 38). The high NPS group exhibited a significantly higher mortality rate compared to the low NPS group, with rates of 33.7% and 8.1%, respectively (*p* < 0.001). A Cox regression analysis indicated that a high NPS score is an independent predictor of one-year mortality, with a hazard ratio of 4.52 (95% CI: 1.93–10.58; *p* < 0.001). **Conclusions:** The NPS is a simple, cheap, and easily accessible tool that can be used for risk stratification and treatment selection in NSTEMI patients. It also highlights the importance of inflammatory and nutritional status in influencing the prognosis of NSTEMI patients.

## 1. Introduction

Non-ST-elevation myocardial infarction (NSTEMI), a critical subtype of acute coronary syndrome, arises from a temporary or incomplete blockage of a coronary artery, resulting in myocardial ischemia without ST-segment elevation on an electrocardiogram. NSTEMI is a prevalent and serious condition with significant implications for both healthcare systems and patient outcomes. Despite advancements in diagnosis and treatment, NSTEMI continues to be associated with significant mortality and morbidity, often exceeding the long-term risk found in ST-elevation myocardial infarction (STEMI), another major form of acute coronary syndrome. Patients with NSTEMI face an elevated risk of heart failure, recurrent ischemic events, and all-cause mortality. Evidence from recent guidelines and population studies highlights the critical need for timely, evidence-based management strategies to mitigate these risks and improve patient prognosis [1,2,3,4,5].

The management of patients diagnosed with NSTEMI necessitates a rapid and precise evaluation of the patient’s clinical presentation and risk profile to guide the selection of optimal treatment strategies. Timely risk stratification is critical for predicting short-term and long-term outcomes, facilitating tailored therapeutic approaches and resource allocation. Several validated risk assessment scores have been created for this purpose, including the TIMI score, the GRACE score, and the CRUSADE score. Each scoring system integrates clinical and laboratory parameters to estimate the probability of adverse events such as mortality, recurrent ischemia, or major bleeding. While the TIMI and GRACE scores focus primarily on ischemic outcomes and mortality, the CRUSADE score incorporates the bleeding risk prediction, offering a complementary perspective in guiding treatment decisions. These tools have been validated across diverse populations and provide invaluable guidance for prognosis and treatment decisions in NSTEMI patients [6,7,8].

The TIMI risk score is a widely recognized and effective tool for stratifying both in-hospital and follow-up mortality risk in patients presenting with NSTEMI. This scoring system incorporates readily available clinical and laboratory variables to provide a simple yet robust prognostic model, enabling clinicians to estimate the likelihood of adverse outcomes and guide therapeutic decisions. Validated through multiple clinical trials and large registries, including the TIMI III registry, the TIMI score has demonstrated consistent reliability in predicting short-term mortality and ischemic events among patients diagnosed with unstable angina or NSTEMI. Its practical application and ease of use have made it a cornerstone in risk stratification for this patient population, ensuring timely intervention and optimized resource allocation [7,9,10].

These risk scores are based on clinical, electrocardiographic, and biochemical variables that reflect the severity and scope of myocardial injury and ischemia. However, these risk scores have some limitations, such as the complexity, lack of validation in contemporary cohorts, and poor discrimination for mortality.

Recent research emphasizes the pivotal role of nutritional status and inflammation as fundamental determinants of prognosis in patients diagnosed with NSTEMI. Inflammation is intricately linked to the underlying mechanisms of atherosclerosis, leading to the rupture of plaques that can trigger NSTEMI episodes. This inflammatory process is not only a driving force behind the progression of atherosclerotic disease but also significantly impacts clinical outcomes, often resulting in adverse events for affected individuals [11,12].

Furthermore, specific inflammatory markers have emerged as important indicators of disease severity and patient prognosis. For example, the platelet/lymphocyte ratio (PLR) and neutrophil/lymphocyte ratio (NLR) are notably linked to an increased severity of coronary artery disease. These markers provide valuable insights and have demonstrated reliability in predicting patient outcomes for those experiencing NSTEMI, thereby aiding in clinical decision-making and management strategies [13,14,15].

The nutritional status is also a great contributor to the outcomes in patients with NSTEMI; malnutrition can often be diagnosed in them and it is associated with higher morbidity and mortality [16]. Simple and widely available indicators, such as serum cholesterol and albumin values, have demonstrated prognostic utility in identifying patients at a higher risk of adverse results. Hypoalbuminemia, in particular, reflects a combination of poor nutritional status and systemic inflammation, contributing to worse survival rates [17]. Similarly, changes in cholesterol levels, a marker of both nutritional and metabolic health, have been linked to long-term mortality, underscoring the importance of comprehensive nutritional assessment in NSTEMI management [18].

The Naples prognostic score (NPS) is a new risk score in which inflammatory and nutritional parameters are combined. Indeed, it comprehensively evaluates the NLR, the lymphocyte/monocyte ratio (LMR), serum albumin, and serum cholesterol levels. First described by Galizia et al., the NPS was intended to determine the postoperative mortality risk after colorectal cancer. Later, the NPS was validated as an independent predictor of both in-hospital mortality and long-term mortality in patients diagnosed with STEMI who underwent primary PCI [19,20].

The TIMI and GRACE risk scores are widely used for predicting mortality in NSTEMI patients. The TIMI score primarily focuses on clinical and electrocardiographic factors, while the GRACE score incorporates additional laboratory parameters and is known for its superior predictive accuracy. However, both scores have limitations, particularly in integrating inflammation and nutritional status, which are crucial factors influencing cardiovascular outcomes. The Naples prognostic score (NPS) differs by incorporating these parameters, making it a potentially more holistic predictor of long-term prognosis in NSTEMI patients. Unlike TIMI and GRACE, which require detailed clinical scoring, the NPS provides a simple and cost-effective alternative that may complement existing risk assessment models in clinical practice. Our hypothesis suggests that the NPS could serve as a simpler yet reliable predictor of one-year mortality in NSTEMI patients undergoing selective PCI. To our knowledge, there has been no prior research evaluating the predictive significance of the NPS within this patient population. Thus, the aim of this study is to investigate the correlation between the NPS and one-year mortality, proposing that the NPS, through its inclusion of inflammatory and nutritional metrics, would offer a more dependable prediction of mortality compared to conventional risk scores.

## 2. Subjects and Methods

### 2.1. Study Design and Population

This study utilized retrospective data from 197 patients diagnosed with NSTEMI between January and December 2020, following approval from the local ethics committee. We screened a total of 238 patients with NSTEMI Patients Undergoing Selective PCI. We excluded 15 patients with a history of previous CABG, 14 patients who were lost to clinical follow-up before one year, and 12 patients with incomplete hospital medical records. Thus, 197 patients were included in the study. The research was conducted in accordance with the Declaration of Helsinki, including the revisions made in 2013.

The demographic, clinical, laboratory, and angiographic characteristics of the patients and their TIMI risk score at admission were obtained from the hospital records. All laboratory values, including albumin, total cholesterol, NLR, and LMR, were measured at the time of hospital admission. The one-year all-cause mortality status of the patients was determined through phone calls or by querying the national death registry system.

The study included participants who met the following criteria: (1) patients diagnosed with NSTEMI, (2) patients who underwent selective PCI, and (3) patients whose TIMI score and NPS ratio could be calculated.

Conversely, individuals were excluded from the study if they met any of the following conditions: (1) patients diagnosed with stable angina pectoris, unstable angina pectoris or STEMI, (2) patients with a history of coronary artery bypass grafting surgery, and (3) patients with missing or incomplete data.

### 2.2. Measurement of the NPS Score

The NPS score was derived by incorporating serum albumin levels, total cholesterol levels, the neutrophil-to-lymphocyte ratio (NLR), and the lymphocyte-to-monocyte ratio (LMR). Following the methodology described by Galizia et al. [21], specific cutoff values for these parameters were determined using MaxStat lite, version 24.3 analysis. Points were assigned to each parameter as follows: a serum albumin level below 40 g/L received 1 point, a total cholesterol level of 180 mg/dL or less received 1 point, an NLR value exceeding 2.96 was given 1 point, and an LMR value of 4.44 or less was assigned 1 point. The total NPS score was calculated by summing these individual scores. The NPS cutoff thresholds, identified using MaxStat analysis in this cohort, were consistent with those validated in previous cardiovascular research. Patients were then categorized into two groups based on their NPS: Group 1 consisted of individuals with an NPS of 0–2, while Group 2 included those with an NPS of 3 or 4. This scoring system facilitated the stratification of patients according to their cardiovascular risk. The one-year mortality encompassed deaths that occurred after being discharged from the hospital.

### 2.3. Laboratory Measurements

Results of laboratory parameters such as complete blood count (CBC), biochemistry panel, and cholesterol panel of all participants were obtained from the hospital data recording system. In our hospital, Coulter Counter LH Series (Beckman Coulter Inc., Miami, FL, USA) is used for CBC and biochemistry panel is evaluated using an automatic chemistry analyzer (Abbott Aeroset, Abbott Park, IL, USA).

### 2.4. Statistical Analysis

The patients’ data were analyzed using IBM SPSS software, version 21.0, to perform statistical evaluations. Continuous variables were expressed as either mean with standard deviation or median with interquartile range, depending on the distribution of the data. Group comparisons were carried out using the independent-samples Student’s *t*-test for continuous variables and Pearson’s chi-square test for categorical variables.

The predictive performance of the NPS score and TIMI risk score was evaluated through receiver operating characteristic (ROC) curve analysis. For each biomarker and risk score, the area under the curve (AUC), along with sensitivity, specificity, positive predictive value (PPV), negative predictive value (NPV), and overall accuracy, was calculated.

Survival curves were constructed using the Kaplan–Meier method and compared using the log-rank test. To identify predictors of mortality, both univariable and multivariable Cox proportional hazards models were employed to estimate hazard ratios (HRs) with 95% confidence intervals (CIs). Variables associated with mortality were initially analyzed using univariable regression, and those with statistically significant *p*-values were subsequently included in the multivariable analysis. A *p*-value of less than 0.05 was considered indicative of statistical significance.

## 3. Results

### 3.1. Baseline Characteristics

The study population included 197 participants with a mean age of 62.9 years, of whom 41.1% were female. There were 111 patients (mean age = 61.34, 36% female) in Group 1 and 86 patients (mean age = 64.98, 48% female) in Group 2. The patients were followed up 12 months after discharge. The overall mortality rate at follow-up was 19.3% (*n* = 38). The mortality rates of patients in Group 2 were found to be statistically significantly higher than those of patients in Group 1 (33.7 vs. 8.1%, respectively, *p* < 0.001). No significant differences were observed between the two groups regarding sex, age, diabetes mellitus, hyperlipidemia, hypertension, or a history of coronary artery disease. Group 2 had higher neutrophil and platelet counts, but lower Hb, RBC, lymphocyte, albumin, and total cholesterol levels than Group 1. Detailed demographic, laboratory and clinical characteristics of the study population according to the NPS are presented in Table 1.

### 3.2. Regression Analysis

Creatinine, hemoglobin, RDW, and the NPS were identified as significant predictors in the univariable Cox regression analysis, each displaying statistically significant *p*-values. The respective hazard ratios (HRs) and 95% confidence intervals (CIs) were as follows: creatinine (HR: 2.56, 95% CI: 1.59–4.13, *p* < 0.001), hemoglobin (HR: 0.80, 95% CI: 0.71–0.90, *p* < 0.001), RDW (HR: 1.18, 95% CI: 1.04–1.34, *p* = 0.007), and the NPS (HR: 4.73, 95% CI: 2.24–10.00, *p* < 0.001). These variables, which demonstrated a strong relationship with mortality risk, were subsequently included in the multivariable regression analysis for further evaluation.

**Figure 1:**
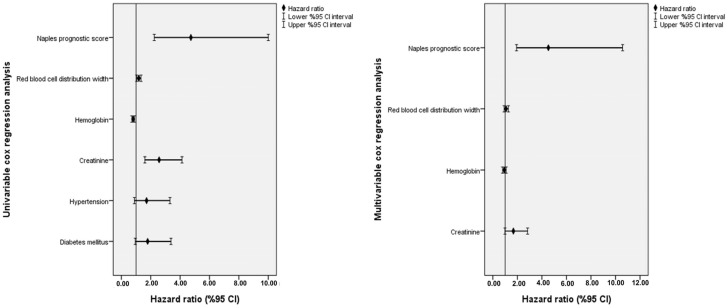
Forest plot graph for the results of univariable and multivariable Cox regression analyses concerning the predictors of one-year mortality.

The multivariable Cox regression analysis confirmed that a high NPS was an independent and robust predictor of one-year mortality, with a hazard ratio of 4.53 (95% CI: 1.94–10.58, *p* < 0.001) (Table 2). This finding underscores the significant prognostic value of the NPS in identifying patients at a greater risk of adverse outcomes within the first year following their diagnosis. To provide a clearer visual representation of the results, the hazard ratios and confidence intervals for the predictors included in the regression analysis were illustrated using a forest plot (Figure 1).

### 3.3. Survival Analysis

**Figure 2:**
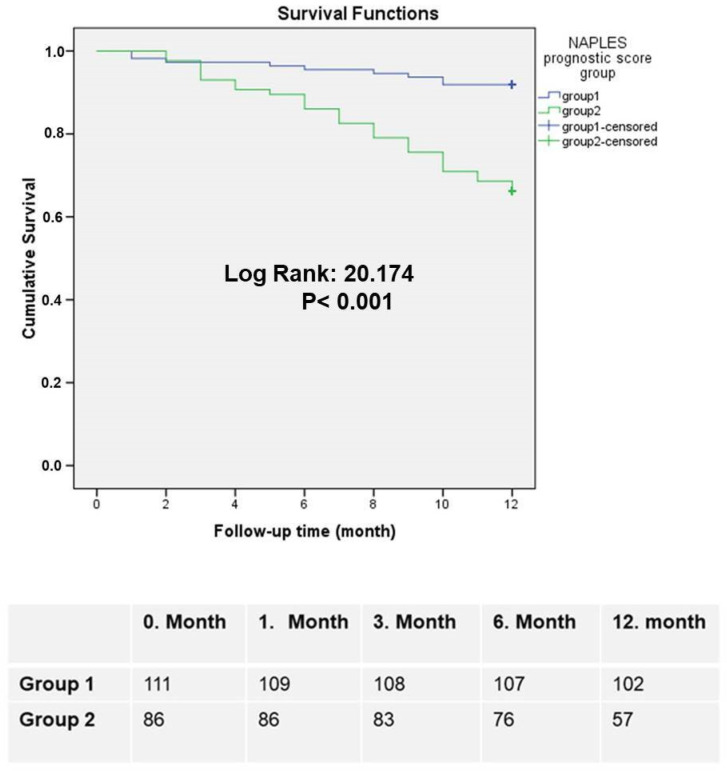
The survival analysis of Kaplan–Meier curve for one-year mortality stratified by the NPS.

In the current study, we showed a Kaplan–Meier survival analysis to explore the association between the elevated NPS values and one-year mortality rates in patients. The results of the Kaplan–Meier analysis demonstrated that patients in the high NPS group had significantly lower survival probabilities at the one-year follow-up compared to those in the low NPS group. This difference was statistically significant, as indicated by the log-rank test result (log-rank: 20.174, *p* < 0.001). These findings emphasize the strong predictive capability of the NPS in assessing the risk of one-year mortality. By clearly distinguishing between patients with high and low survival probabilities, the Kaplan–Meier analysis further underscores the prognostic utility of the NPS as a valuable tool for stratifying patients based on their likelihood of survival. A graphical representation of the survival curves provides additional visual confirmation of this trend, as shown in Figure 2.

### 3.4. The Analysis of Receiver Operator Characteristic Curve

**Figure 3:**
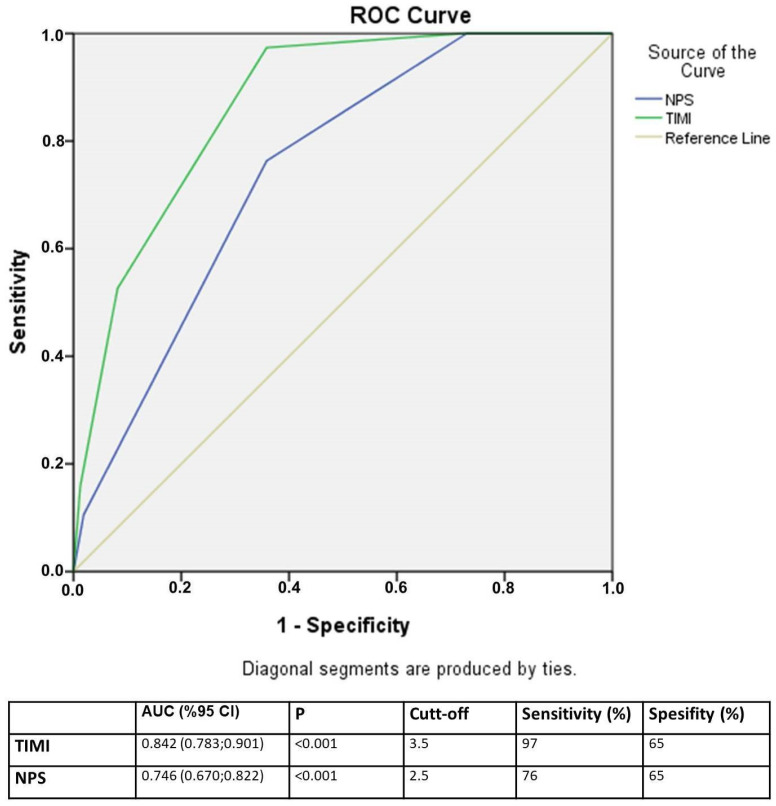
The analysis of receiver operator characteristic curve.

A receiver operating characteristic curve analysis was conducted to assess the ability of the NPS in predicting one-year mortality. The analysis revealed that the NPS exhibited strong prognostic accuracy, as evidenced by an AUC of 0.746 (95% CI: 0.670–0.822, *p* < 0.001), indicating a reliable capacity to distinguish between patients at high and low risk of mortality. The optimal cut-off value for the NPS score was determined to be 2.5, which provided a sensitivity of 76% and a specificity of 65%, signifying a good balance between correctly identifying high-risk patients and minimizing false positives. These findings, graphically presented in Figure 3, underscore the clinical utility of the NPS in stratifying patients based on their one-year mortality risk.

## 4. Discussion

This study was designed to investigate the association between the NPS and one-year mortality in NSTEMI patients undergoing selective PCI. We investigated the link between the NPS and one-year clinical outcomes, offering valuable insights into its importance in risk assessment. This indicates that the NPS is not only a simple and accessible tool but also highly effective in risk stratification and treatment selection for NSTEMI patients. These findings further support the growing evidence for including inflammatory and nutritional markers in cardiovascular risk assessment models.

As far as we know, this represents the first analysis examining the prognostic performance of the NPS exclusively in patients presenting with NSTEMI. Our observations confirm the consistency with previously conducted investigations into the performance of the NPS in several clinical and demographic groups. It has been shown to predict short- and midterm mortality and rehospitalization rates in patients with heart failure, underlining the value of the NPS as a prognostic tool in this population [19]. In addition, in patients with STEMI, the NPS has been found to be strongly associated with long-term mortality, further documenting the utility of this tool in cardiovascular risk stratification [20]. Furthermore, the NPS has shown a significant correlation with in-hospital mortality among HF patients, highlighting its potential to predict adverse outcomes and guide clinical management [22]. In STEMI populations, the NPS has also been independently linked to in-hospital adverse events and all-cause mortality during follow-up, providing evidence of its predictive strength for both short-term and long-term outcomes [19]. Apart from cardiovascular disorders, the NPS has also proven effective in postoperative complication prediction in patients with colectomy for diverticulitis, and, thus, it has a broader application as a marker of systemic inflammation and nutritional status [23].

In a study involving 325 patients, the NPS was shown to be a reliable and effective tool for predicting 30-day all-cause mortality in individuals diagnosed with an acute pulmonary embolism [24]. Furthermore, Pay et al. demonstrated that the NPS has substantial potential in forecasting long-term mortality in patients with an acute pulmonary embolism, highlighting its value in both short- and extended-term risk assessments [25]. Additionally, research has identified the NPS as an independent and superior predictor of major adverse cardiac events in patients with chronic kidney disease undergoing percutaneous coronary intervention, emphasizing its relevance in this high-risk population [26]. In a comprehensive study of 860 patients, the NPS was found to be a significant and independent predictor of postoperative atrial fibrillation in individuals undergoing cardiac surgery, particularly those of advanced age, further reinforcing its versatility as a prognostic marker [27]. Another investigation, which included 222 patients diagnosed with severe aortic stenosis who are undergoing transcatheter aortic valve implantation, concluded that the NPS is a valuable tool for predicting one-year mortality as well as major adverse cardiovascular events, thereby demonstrating its utility in patients with structural heart disease [28]. Çetin et al. also showed that the NPS is a predictor of mortality in TAVI patients [29]. Uysal et al. found that the NPS effectively predicts in-hospital mortality in acute ischemic stroke patients undergoing endovascular treatment, with higher scores associated with increased mortality rates [30]. Dervis et al. demonstrated that the NPS is an independent predictor of long-term mortality in patients with chronic limb-threatening ischemia undergoing endovascular revascularization for below-the-knee lesions [31].

The NPS ratio reflects both inflammatory and nutritional status, which are important determinants of the prognosis of NSTEMI patients. Inflammation is a very important component of the pathophysiology of atherosclerosis and plaque rupture that leads to NSTEMI [6,7]. Nutritional status is another aspect that influences the clinical outcomes of NSTEMI patients. The presence of malnutrition is a common condition in NSTEMI patients and has been associated with an increased risk of mortality and morbidity. Therefore, inflammatory and nutritional status indicators, such as the NLR, PLR, systemic immune and inflammation index, Controlling Nutritional Status, Geriatric Nutritional Risk Index, Prognostic Nutritional Index, etc., have been investigated in NSTEMI patients and have been shown to be associated with mortality [11,12,13,14,15,16,17].

The coexistence of malnutrition and inflammation amplifies the risk of adverse outcomes, suggesting that these factors may have a synergistic effect on prognosis. Nutritional interventions could potentially improve outcomes, although further research is needed to confirm this hypothesis. While the studies emphasize the negative impact of a poor nutritional and inflammatory status on NSTEMI outcomes, they also suggest potential areas for intervention. Addressing malnutrition and controlling inflammation could be key strategies in improving patient prognosis, particularly in the elderly and those with elevated inflammatory markers.

Our study demonstrates that the NPS ratio is a parameter that can capture the complex interaction of inflammatory and nutritional factors that affect the prognosis of NSTEMI patients.

This study indicates that the NPS ratio is a simple, cost-effective, and easily accessible measure that can be used for risk stratification and treatment selection in patients with NSTEMI. Unlike the TIMI and GRACE scores, which primarily focus on myocardial injury and ischemia, the NPS takes into account inflammatory and nutritional factors. This comprehensive approach provides a well-rounded risk profile that can be particularly useful for guiding personalized treatments for NSTEMI patients. Clinically, the NPS could help prioritize high-risk patients for early interventions, given its simplicity and ease of use in routine practice.

Future research is needed to validate these findings in prospective, multicenter trials. Moreover, the NPS ratio should be compared with other risk scores, and inflammatory and nutritional status indicators in terms of predicting mortality in NSTEMI patients. Finally, the effect of the NPS ratio on predicting mortality should be examined for the subgroups of mortality (such as cardiac mortality, sudden cardiac death, mortality due to heart failure, etc.) in NSTEMI patients.

Limitations include the fact that our findings have to be judged with the retrospective study design and a small sample size, which prevented generalization for wider patient groups. Another limitation could be the incomplete data of the patients since their exclusion may cause a selection bias whose extent cannot be precisely estimated. Another important shortcoming is the lack of an accurate correlation analysis of the NPS with other inflammatory markers or nutritional indices, since this could provide further insight into the real prognostic value. The evaluation of our series was carried out considering only the NPS value upon admission. This method has the advantage of yielding very valuable information, but it does not consider the possible dynamic variations in the albumin level, hematological parameters, and overall nutritional status in the follow-up period. Dehydration on admission and temporal changes in nutritional and inflammatory markers may have been operating during NPS estimation, thus affecting the outcomes. These limitations highlight the need for further investigations on larger cohorts, prospectively designed with more detailed evaluations of longitudinal variations in clinical parameters to confirm and extend our findings.

## 5. Conclusions

The NPS is a valuable and independent predictor of one-year mortality in NSTEMI patients undergoing selective PCI. It effectively integrates crucial inflammatory and nutritional factors, delivering a thorough risk assessment for patients. The NPS offers a simple, cost-effective, and easily accessible tool for clinicians to enhance risk stratification and guide treatment decisions in NSTEMI patients. Large-scale prospective studies are warranted to validate these findings and assess the potential benefits of incorporating NPS into routine clinical practice for managing NSTEMI patients.

## Figures and Tables

**Table 1:** Baseline characteristics, laboratory findings, and mortality rates according to Naples prognostic score groups in patients with NSTEMI (*n* = 197).

Variables	Naples Prognostic Score			
	Overall (*n* = 197)	Group 1 (*n* = 111)	Group 2 (*n* = 86)	*p*
Age, years	62.93 (13.55)	61.34 (13.28)	64.98 (13.69)	0.063 ^1^
Gender (female), *n* (%)	81 (41)	40 (36)	41 (48)	0.110 ^2^
Diabetes mellitus, *n* (%)	72 (36)	39 (35)	33 (38)	0.657 ^2^
Hypertension, *n* (%)	101 (51)	56 (50)	45 (52)	0.886 ^2^
Hyperlipidemia, *n* (%)	25 (13)	18 (16)	7 (8)	0.130 ^2^
History of CAD; *n* (%)	85 (43)	46 (41)	39 (45)	0.664 ^2^
Glucose, mg/dL	166.80 (82.85)	168.49 (83.60)	164.60 (82.31)	0.744 ^1^
Ure, mg/dL	44.31 (25.52)	40.02 (20.20)	49.85 (30.31)	0.010 ^1^
Creatinine, mg/dL	1.05 (0.45)	0.97 (0.33)	1.15 (0.57)	0.012 ^1^
Albumin, g/dL	41.85 (3.85)	42.48 (3.80)	41.05 (3.79)	0.009 ^1^
AST, U/L	36.31 (19.0–35.0)	36.57 (19.0–35.0)	35.96 (20.0–35.7)	0.911 ^1^
ALT, U/L	29.33 (14.0–30.0)	30.57 (14.0–34.0)	27.74 (13.7–29.0)	0.559 ^1^
Total cholesterol, mg/dL	163.56 (41.33)	170.66 (42.82)	154.40 (37.62)	0.005 ^1^
Triglycerides, mg/dL	132.17 (66.42)	134.14 (60.75)	129.62 (73.38)	0.645 ^1^
LDL-C, mg/dL	108.24 (28.23)	112.26 (30.31)	103.05 (25.80)	0.022 ^1^
HDL-C, mg/dL	45.48 (9.26)	44.29 (89.48)	47.03 (8.78)	0.037 ^1^
WBC, ×10^3^/μL	9.49 (3.43)	9.35 (3.33)	9.67 (3.58)	0.522 ^1^
Hemoglobin, g/dL	13.75 (3.32)	14.36 (3.79)	12.95 (2.39)	0.002 ^1^
Hematocrit, %	40.67 (6.58)	42.05 (6.10)	38.89 (6.77)	0.001 ^1^
Platelet count, ×10^3^/μL	246.98 (83.01)	238.18 (72.11)	258.35 (94.50)	0.102 ^1^
RDW	14.36 (1.81)	14.18 (1.69)	14.59 (1.93)	0.129 ^1^
Lymphocyte, 10^3^/μL;	2.02 (1.14–2.58)	2.52 (1.75–3.12)	1.39 (0.92–1.85)	<0.001 ^1^
Neutrophils, 10^3^/μL;	6.34 (4.39–7.86)	5.85 (4.12–7.04)	6.97 (5.15–8.91)	0.002 ^1^
Monocytes, 10^3^/μL	0.61 (0.42–0.70)	0.60 (0.41–0.70)	0.63 (0.46–0.71)	0.717 ^1^
TIMI risk score	2.21 (0.92)	3.05 (0.93)	3.99 (1.07)	<0.001 ^1^
Mortality, *n* (%)	38 (19.3)	9 (8.1)	29 (33.7)	<0.001 ^2^

^1^ Student’s *t*-test *p*-value; ^2^ Pearson’s chi-square test *p*-value; comparison of baseline characteristics by predicted group assignment. *p* < 0.05 was considered statistically significant. Values are presented as *n* (%) or median (interquartile range) or mean (standard deviation), depending on the variable distribution. Abbreviations: CAD, coronary artery disease; ALT, alanine aminotransferase; AST, aspartate aminotransferase; HDL-C, high-density lipoprotein cholesterol; LDL-C, low-density lipoprotein cholesterol; WBC, white blood cell; RDW, red blood cell distribution width; TIMI, the thrombolysis in myocardial infarction (TIMI) risk score.

**Table 2:** Univariable and multivariable Cox regression analyses for the independent prediction of one-year mortality.

Parameters	Univariable Analysis		Multivariable Analysis	
	HR (%95 CI)	*p*-Value	HR (%95 CI)	*p*-Value
Diabetes mellitus	1.787 (0.946–3.376)	0.074		
Hypertension	1.714 (0.887–3.314)	0.109		
Creatinine	2.569 (1.599–4.127)	<0.001	1.673 (0.988–2.833)	0.055
Hemoglobin	0.803 (0.714–0.904)	<0.001	0.930 (0.794–1.089)	0.366
RDW	1.185 (1.048–1.340)	0.007	1.075 (0.914–1.265)	0.383
Naples prognostic score	4.731 (2.238–10.000)	<0.001	4.525 (1.935–10.581)	<0.001

*p* < 0.05 was considered statistically significant. Abbreviations: 95% CI, 95% confidential interval; HR, hazard ratio; RDW, red blood cell distribution width.

## Data Availability

All the data collected or analyzed in this study are included in this article.
